# Influence of Obesity and Exercise on β2-Adrenergic-Mediated Anti-Inflammatory Effects in Peritoneal Murine Macrophages

**DOI:** 10.3390/biomedicines8120556

**Published:** 2020-11-30

**Authors:** Leticia Martín-Cordero, Isabel Gálvez, María Dolores Hinchado, Eduardo Ortega

**Affiliations:** 1 Instituto Universitario de Investigación Biosanitaria de Extremadura (INUBE), 06071 Badajoz, Spain; leticiamartin@unex.es (L.M.-C.); igalvez@unex.es (I.G.); mhinsan@unex.es (M.D.H.); 2 Grupo de Investigación en Inmunofisiología, Departamento de Enfermería, Centro Universitario de Plasencia, Universidad de Extremadura, 10600 Plasencia, Spain; 3 Grupo de Investigación en Inmunofisiología, Departamento de Enfermería, Facultad de Medicina, Universidad de Extremadura, 06071 Badajoz, Spain; 4 Grupo de Investigación en Inmunofisiología, Departamento de Fisiología, Facultad de Ciencias, Universidad de Extremadura, 06071 Badajoz, Spain

**Keywords:** obesity, M1 and M2 macrophages, regular exercise, terbutaline, inflammation, cytokines

## Abstract

Obesity is a chronic low-grade inflammatory condition, and β2-adrenergic agonists as well as exercise have been proposed as anti-inflammatory strategies in obesity, so it is critical to accurately determine the effects of β2-adrenergic stimulation, especially when combined with other non-pharmacological therapies. The aim of this investigation was to determine the effect of β2-adrenergic activation on the inflammatory profile and phenotype of macrophages, and whether these effects could be affected by obesity and exercise in this condition. High-fat diet-induced obese and lean C57BL/6J mice were allocated to sedentary or exercised groups. The inflammatory profiles and phenotypes of their peritoneal macrophages were assessed by flow cytometry in the presence or absence of the selective β2-adrenergic receptor agonist terbutaline. β2-adrenergic activation caused global phenotypic anti-inflammatory effects in lean and obese sedentary mice, which were more drastic (also including anti-inflammatory effects on the cytokine profile) in obese animals. In exercised lean and obese animals, this anti-inflammatory effect is weaker and only evident by decreased iNOS and IL-8 expression, without changes in the anti-inflammatory markers. Therefore, β2-adrenergic activation leads to anti-inflammatory effects, but these effects are modulated by obesity in sedentary conditions, as well as by regular exercise; but not by obesity in trained conditions.

## 1. Introduction

Obesity is considered a low-grade inflammatory condition, that is, a chronic systemic condition involving elevated systemic concentrations of inflammatory cytokines such as tumor necrosis factor-alpha (TNF-α), interleukin (IL)-1β, IL-6, IL-1RA, and other inflammatory mediators such as C-reactive protein (CRP) [1,2,3,4,5,6]. Inflammatory cytokines are able to stimulate the hypothalamus-pituitary-adrenal (HPA) axis, leading to increased levels of glucocorticoids that affect several inflammatory and immune processes, and disruptions of this cytokine-HPA axis feedback loop can aggravate inflammatory pathologies [7]. In obesity, alterations of the HPA axis and the sympathetic nervous system (SNS) function are also implicated in its pathophysiology [8]. Therefore, anomalies in the immune and inflammatory activities in this condition are also accompanied by altered neuroendocrine responses and dysregulated feedback mechanisms between the immune and stress responses [8,9,10].

Catecholamines secreted by the SNS and the adrenal glands are endogenous adrenergic agonists that are crucial in regulating metabolism as well as most of the immune response mechanisms, including systemic and local release of inflammatory cytokines and chemokines and the innate response [7,11,12,13,14,15,16]. Therefore, adrenergic regulation of the innate immune response via β2-adrenergic receptors could be a novel anti-inflammatory and immunomodulating target with therapeutic potential [17,18,19], particularly as an anti-inflammatory strategy in the management of obesity.

Macrophages are typically divided into two groups based on whether they are classically (pro-inflammatory, M1) or alternatively activated (anti-inflammatory, M2). M1 macrophages are linked to cellular immunity and microbicidal activity as well as production of pro-inflammatory mediators; whereas M2 macrophages are associated with production of anti-inflammatory mediators, tissue repair and remodeling processes, and humoral immunity [20,21,22]. In obesity, macrophages presenting a M1 phenotype (expression of inducible nitric oxide synthase, iNOS) are more prevalent than macrophages with M2 phenotype (expression of type-1 arginase, ARG1) [20,21,22]. Similarly, obese individuals show higher prevalence of circulating monocytes with a pro-inflammatory phenotype and activity profile [23]. Thus, it is pivotal to explore macrophage polarization in response to β2-adrenoceptor stimulation in obesity, focusing on determining if this response is the same or different in obese and healthy lean individuals.

Furthermore, it has been proposed that anti-inflammatory effects such as the increase in catecholamine levels and a potential decrease in the percentage of cells with an inflammatory profile could be key factors mediating the beneficial effects of regular exercise, beneficial effects that are particularly relevant in obese individuals [8,24,25,26]. During exercise, immune-neuroendocrine responses involving the HPA axis, the SNS, and macrophages can be different in healthy individuals, in patients with inflammatory pathologies, and/or after pathogen challenge. This regulation by exercise depends on each individual’s basal set-point, being anti-inflammatory mainly (or only) in the case of a highly inflammatory status [27]. Particularly in macrophages, the aim of exercise as an anti-inflammatory strategy is to achieve good transitions between M1 and M2 macrophages [27]. Therefore, regular exercise can induce anti-inflammatory benefits by switching the inflammatory phenotype of monocytes and macrophages in obese individuals [24,26]. This way, exercise programs must be aimed at achieving a decrease in dysregulated levels of inflammatory mediators together with optimal phenotypic transitions between M1 and M2 macrophages [8,16].

Bearing this in mind, it is very important to accurately determine the effects of β2-adrenergic stimulation, and to avoid potential adverse or undesired effects of anti-inflammatory pharmacological strategies via stimulation of β2-adrenergic receptors, especially when combined with other anti-inflammatory strategies such as physical exercise. Thus, the first objective of this study was to determine the effect of β2-adrenergic activation on the inflammatory profile and phenotype of macrophages, and whether these effects could be affected by obesity. The second objective was to know if regular exercise could modify the effects of β2-adrenergic stimulation on the inflammatory profile and phenotype of macrophages, especially in obese animals. To the best of our knowledge, this is the first investigation to comprehensively analyze the influence of exercise and obesity in β2-adrenergic regulation of the inflammatory profile of macrophages.

## 2. Experimental Section

### 2.1. Experimental Design

This investigation is part of a greater research project (DEP2015-66093-R) focused on evaluating the effects of β2-adrenergic modulation of the innate and inflammatory responses in obesity and exercise. 22 C57BL/6J mice (Envigo, Huntingdon, UK) were randomly allocated to one of two diets at eight weeks of age until sacrifice 18 weeks later. One of the groups (*n* = 11) (obese group) was placed on a high-fat diet (HFD) (260HF diet; SAFE, Augy, France) containing 36% fat (58.8% of the energy from fat), which is optimal for the study of obesity and its complications in mice [23]. The other group (*n* = 11) was placed on standard laboratory rodent chow (SD) (A04 diet; SAFE, Augy, France), containing 3.1% fat (8.4% of the energy from fat), constituting the healthy control group (lean group). Each group was randomly divided into two sub-groups, sedentary and trained groups.

Mice had free access to food and water and were housed individually, in a temperature- and humidity-controlled room (22 ± 1 °C; 60 ± 5%) with a 12  h light/12  h dark cycle (23:00–11:00 h light; 11:00–23:00 h dark).

After 10 weeks of diet protocol, the group of obese trained mice (*n* = 5) and the group of lean trained mice (*n* = 5) were subjected to a protocol of habitual exercise for 8 weeks. After 12 h fasting and 72 h of rest for the trained groups, peritoneal suspension and blood samples were collected from anaesthetized animals.

The study was approved by the Bioethics Committee for Animal Experimentation of the University of Extremadura (registry numbers 115/2015 for project DEP2015-66093-R, July 2015; 70/2018 for project IB18011, July 2018), in accordance with the National and European legislation for the protection of animals used for research.

### 2.2. Exercise

Habitual exercise training was carried out 3 days per week for 8 weeks, always at the same time in the active period (dark 11:00–23:00 h). Animals performed treadmill running (model 800, IITC Life Science Inc., Los Angeles, CA, USA) with no slope, with intensity and duration progression from 10 m/min for 10 min in the first week to 18 m/min for 45 min in the last week. Animals were culled 72 h after the last training session.

### 2.3. Collection of Biological Samples, Cell Culture and Incubation

Fasted animals were gas anaesthetized with isoflurane, by standard procedure (starting dose 3–5% isoflurane, maintenance dose 1.5–3% isoflurane). Biological samples were obtained from live, anaesthetized animals. Whole blood was drawn by cardiac puncture. Fasting blood glucose concentration and lipid profile including total cholesterol, high-density lipoprotein cholesterol (HDL-C), calculated low-density lipoprotein cholesterol (cLDL-C) and triglycerides (TG) were measured in whole blood (LUX^®^, Biochemical Systems International Srl, Arezzo, Italy) [23,26,28].

Peritoneal suspension was obtained by injecting (and then extracting) 4 mL of phosphate buffered saline (PBS) into the peritoneal cavity. Peritoneal suspension was adjusted to 10^6^ cells/mL in RPMI 1640 complete medium (L-glutamine and penicillin-streptomycin) (Thermo Fisher Scientific, Waltham, MA, USA) without fetal bovine serum (FBS), following standard procedures previously carried out in our laboratory [9,11,28]. Cells were cultured with 1 μg/mL brefeldin A solution (Thermo Fisher Scientific), in order to inhibit protein transport and enhance intracellular staining of cytokines, in the presence or absence of the selective β2 adrenergic receptor agonist terbutaline (1 μM) (Sigma-Aldrich, St. Louis, MO, USA). Plates were incubated for 5 h at 37 °C, 5% CO_2_.

### 2.4. Inflammatory Biomarkers Expression Assays by Flow Cytometry

Samples were centrifuged and cells were incubated (25 min in darkness and agitation) with 600 µL of staining buffer [cold PBS solution plus 0.5% bovine serum albumin (BSA) and 2 mM EDTA (Thermo Fisher Scientific)] plus 750 µL of Inside Fix reagent from Inside Stain Kit (Miltenyi Biotec, Bergisch Gladbach, Germany) to fix cells for intracellular staining. Samples were then centrifuged, and pellets were resuspended in 300 µL of staining buffer. After an overnight incubation (4 °C), samples were centrifuged and pellets were resuspended in Inside Perm reagent (300 µL) from Inside Stain Kit (Miltenyi Biotec) to permeabilize cells for intracellular staining.

A volume of 50 µL of the suspension per well (96-well plate) was dispensed. Cells were incubated with the respective conjugated antibodies for the evaluation of the intracellular expression of inducible nitric oxide synthase (iNOS) (iNOS antibody 4E5, Novus Biologicals, Centennial, CO, USA, NBP2-22119), ARG1 (ARG1 PE, Novus Biologicals, NBP1-32731PE), MCP-1 (Anti-CCL2-PE, Miltenyi Biotec, REA485), TNF-α (Anti-TNF-α-FITC, Miltenyi Biotec, REA636), IL-8 (CXCR1/IL-8 RA APC, Novus Biologicals, FAB8628A), IL-6 (Anti-IL-6-PE, Miltenyi Biotec, REA1034), IL-10 (Anti-IL-10-APC, Miltenyi Biotec, JES5-16E3), and TGF-β (LAP PE-Cyanine7, Thermo Fisher Scientific, TW7-16B4) in macrophages. Briefly, iNOS antibody was incubated first, for 30 min in darkness and agitation. Then, cells were washed and incubated with the conjugated secondary antibody (Alexa Fluor 430 anti-mouse, Thermo Fisher Scientific, AB_2534110.) for 30 min. After another wash, the rest of antibodies were added and incubated for 20 min in the dark with shaking. Finally, plates were centrifuged, supernatants were removed, and 100 µL of Inside Perm reagent were added to each well.

Samples were analyzed by a flow cytometer (CytoFLEX S, Beckman Coulter Life Sciences, Indianapolis, IN, USA), with a minimum of 5000 cells acquired by well. Macrophage population was gated by FSC/SSC parameters and data were processed using the CytExpert software (Beckman Coulter Life Sciences).

### 2.5. Statistical Analysis

Values are expressed as mean ± standard error of the mean (SEM). The variables were normally distributed (tested by the Kolmogorov–Smirnov normality test). Student’s t-test was used for comparisons between groups (paired samples). Minimum significance level was set at *p* < 0.05.

## 3. Results

### 3.1. Animal Model of Obesity

Recently published investigations have already shown that this HFD protocol causes obesity in this mouse strain: mice present high fasting glucose concentration (>250 mg/dL as the recommended threshold [29]) and elevated levels of triglycerides (TG), total cholesterol, high-density lipoprotein cholesterol (HDL-C), and calculated low-density lipoprotein cholesterol (cLDL-C). They have also shown that this protocol of habitual exercise decreases TG, HDL-C, and cLDL-C levels in obese and lean animals [27,28]. All of these results are in line with those obtained in the present work, since the same animals and protocols were used. It is important to note that these values were obtained in anaesthetized animals.

Results regarding body weight, dietary and energy intake, and metabolic parameters in lean and obese mice, both sedentary and exercised, are presented in Table 1.

### 3.2. Influence of Obesity and Exercise in this Condition on the β2 Adrenergic Regulation of the Inflammatory Profile of Peritoneal Macrophages

**Figure 1:**
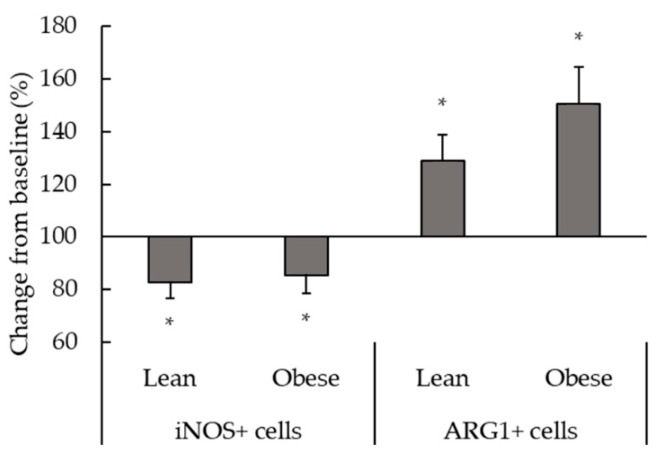
Effect of β2-adrenergic stimulation by terbutaline on the expression of pro-inflammatory phenotype marker iNOS and the anti-inflammatory phenotype marker ARG1 in peritoneal macrophages from sedentary obese and lean mice. Values are expressed as percentage change from baseline, giving the value “100” to the basal values in absence of terbutaline. Each column shows the mean ± SEM of 6 independent assays performed in duplicate. * *p* < 0.05 vs. the corresponding control values without terbutaline (Student’s t-test).

Figure 1 shows the results corresponding to the effect of terbutaline on the inflammatory phenotype of macrophages from lean and obese animals. Terbutaline-mediated β2-adrenergic stimulation increased the expression of ARG1 and decreased iNOS in peritoneal macrophages in both obese and lean sedentary animals, reflecting a transition towards an anti-inflammatory phenotype (M2) in macrophages in response to terbutaline (Figure 1).

**Figure 2:**
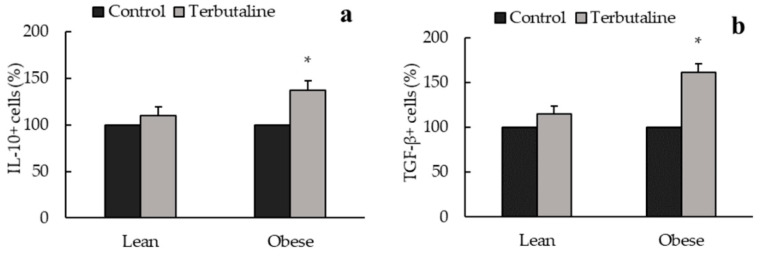
β2-adrenergic stimulation by terbutaline on anti-inflammatory cytokines expression in peritoneal macrophages from sedentary obese and lean mice: IL-10 (**a**) and TGF-β (**b**). Values are expressed as percentage change from baseline, giving the value “100” to the basal (control) values in absence of terbutaline. Each column shows the mean ± SEM of 6 independent assays performed in duplicate. * *p* < 0.05 vs. the corresponding control values without terbutaline (Student’s t-test).

**Figure 3:**
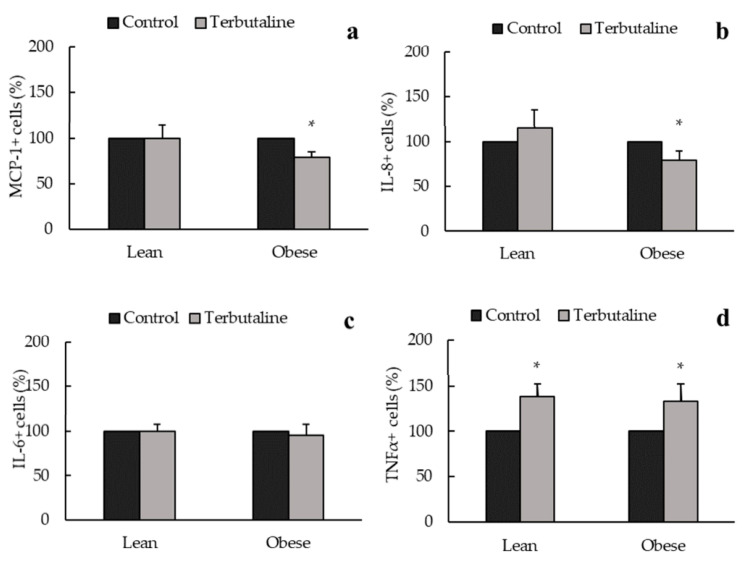
β2-adrenergic stimulation by terbutaline on pro-inflammatory cytokines expression in peritoneal macrophages from sedentary obese and lean mice: MCP-1 (**a**), IL-8 (**b**), IL-6 (**c**) and TNF-α (**d**). Values are expressed as percentage change from baseline, giving the value “100” to the basal (control) values in absence of terbutaline. Each column shows the mean ± SEM of 6 independent assays performed in duplicate. * *p* < 0.05 vs. the corresponding control values without terbutaline (Student’s t-test).

In addition, only in obese animals, β2-adrenergic activation increased intracellular expression of anti-inflammatory cytokines IL-10 (Figure 2a) and TGF-β (Figure 2b) and decreased pro-inflammatory cytokines IL-8 (Figure 3a) and MCP-1 (Figure 3b) expression in peritoneal macrophages, reinforcing the anti-inflammatory effect of terbutaline in these cells in obesity. No terbutaline-induced changes were observed in IL-6 expression (Figure 3c) and surprisingly, β2 adrenergic stimulation increased TNF alpha expression in both lean and obese animals (Figure 3d).

**Figure 4:**
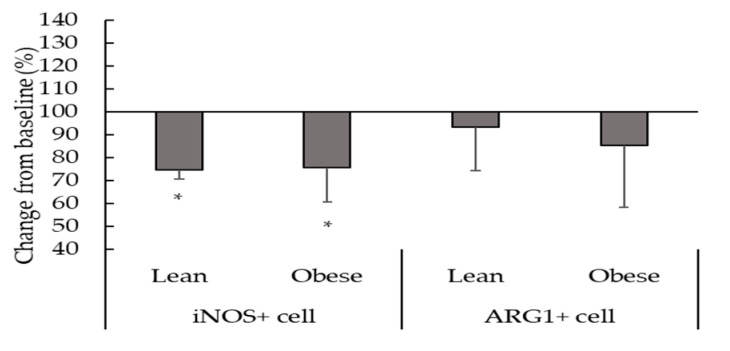
Effect of β2-adrenergic stimulation by terbutaline on the expression of pro-inflammatory phenotype marker iNOS and the anti-inflammatory phenotype marker ARG1 in peritoneal macrophages from trained obese and lean mice. Values are expressed as percentage change from baseline, giving the value “100” to the basal values in absence of terbutaline. Each column shows the mean ± SEM of 5 independent assays performed in duplicate. * *p* < 0.05 vs. the corresponding control values without terbutaline (Student’s t-test).

**Figure 5:**
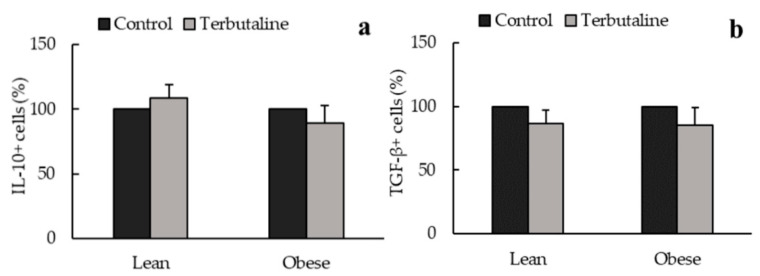
β2-adrenergic stimulation by terbutaline on anti-inflammatory cytokines expression in peritoneal macrophages from trained obese and lean mice: IL-10 (**a**) and TGF-β (**b**). Values are expressed as percentage change from baseline, giving the value “100” to the basal (control) values in absence of terbutaline. Each column shows the mean ± SEM of 5 independent assays performed in duplicate.

**Figure 6:**
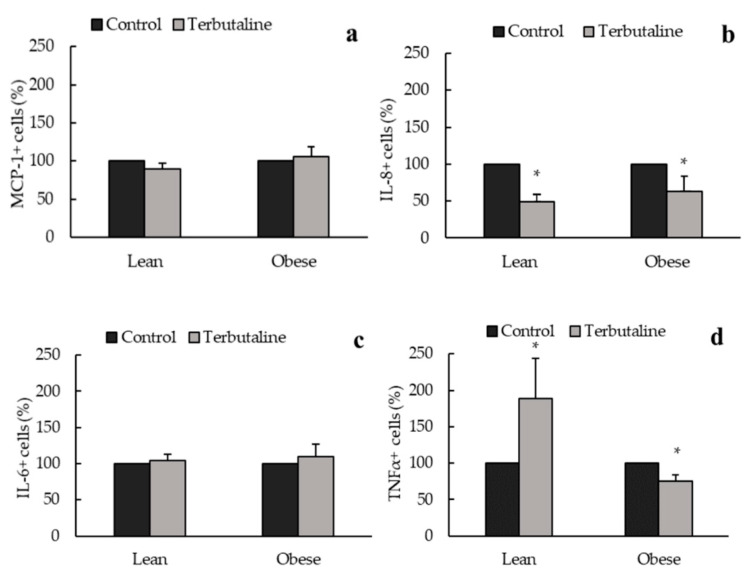
β2-adrenergic stimulation by terbutaline on pro-inflammatory cytokines expression in peritoneal macrophages from trained obese and lean mice: MCP-1 (**a**), IL-8 (**b**), IL-6 (**c**) and TNF-α (**d**). Values are expressed as percentage change from baseline, giving the value “100” to the basal (control) values in absence of terbutaline. Each column shows the mean ± SEM of 5 independent assays performed in duplicate. * *p* < 0.05 vs. the corresponding control values without terbutaline (Student’s t-test).

Figure 4, Figure 5 and Figure 6 show the effect of β2-adrenergic activation on the inflammatory phenotype and inflammatory cytokine expression in macrophages from exercised animals. After the regular physical exercise program, terbutaline decreased the expression of pro-inflammatory phenotype marker iNOS (Figure 4) and the pro-inflammatory cytokine IL-8 (Figure 6b) in peritoneal macrophages from both obese and lean mice. However, no differences were found in the intracellular expression of ARG1 (Figure 4), IL-10, TGF-β (Figure 5a,b), MCP-1 and IL-6 (Figure 6a,c) after β2-adrenergic stimulation. In trained animals, however, terbutaline caused an even greater increase of TNF-α in lean mice; but in obese mice terbutaline seems to revert terbutaline’s stimulatory effect by decreasing the expression of TNF-α after regular physical exercise (Figure 6d).

## 4. Discussion

It is well-known that a pro-inflammatory status together with a dysregulated innate response underlies obesity [1,2,3,4,5,23]. C57BL/6J mice fed HFD develop well-stablished type 2 diabetes mellitus and tissue inflammation [29]. In fact, in the model of obesity used in the present work (C57BL/6J with 60% HFD) mice present systemic inflammation, also reflected by a pro-inflammatory phenotype and cytokine profile of circulating monocytes [23]. However, it is important to note that peritoneal macrophages do not seem to present a higher inflammatory profile in these mice. It can be hypothesized that greater tissue infiltration of macrophages with a pro-inflammatory profile leaves less activated cells in the peritoneal cavity. In fact, recent results have shown that adipose tissue from these obese animals present higher macrophage infiltration and presence of inflammatory structures compared to lean animals [30,31]. The beneficial effect of exercise on this pathophysiological condition is importantly mediated by its anti-inflammatory properties, that are at least partly mediated via adrenergic regulation [16,24]. Different β adrenergic receptors subtypes are expressed in different tissues and cells. Although favorable metabolic effects induced by selective β3 adrenergic receptor stimulation have been also proposed for the treatment of type 2 diabetes and obesity, this receptor is located fundamentally in the adipose tissue [32,33], while β2 adrenergic receptors are largely expressed in different immune cell types, including macrophages [34,35,36]. β2-adrenergic agonists have also been proposed as a potential pharmacological anti-inflammatory strategy in obesity-induced diabetes and its related complications [18,19], targeting monocytes/macrophages activation in the treatment of diabetic complications. Particularly, it has been suggested that the protective action of β2 adrenergic receptor agonists involves abolishing the recruitment of M1-activated macrophages into the renal and cardiac tissues rather than induction of a phenotypic switch from M1 to M2 [18]. Thus, although β2-adrenergic agonists usually inhibit the release of pro-inflammatory cytokines by macrophages, in some specific local responses and under certain circumstances, such as stress, they may stimulate inflammatory responses through the release of inflammatory cytokines [12]. Therefore, it is an interesting issue to ascertain the inflammatory response caused by β2-adrenergic stimulation in obese individuals both sedentary and performing regular exercise, in order to determine whether obesity and exercise in this condition are factors that can influence this adrenergic regulation.

Changes in the recruitment and activation of macrophages have been suggested to crucially contribute to the regulation of metabolic homeostasis, with a proposed pathogenic role for M1 and a protective role for M2 in experimental models of obesity and metabolic disease [21]. Moreover, macrophage polarization from M1 to M2 could potentially provide a potential target of intervention for inflammation and insulin resistance in obesity [37]. Enzymes iNOS and ARG1 expression reflect the route of arginine metabolism within the cell and can also be used to ascertain the inflammatory phenotype of monocytes/macrophages in rodents and humans [38]. Results presented in this work showed that terbutaline-mediated β2-adrenergic stimulation activates polarization of peritoneal macrophages towards anti-inflammatory M2 macrophages (since there is an increase of intracellular expression of ARG1), to the detriment of pro-inflammatory M1 macrophages (there is decreased iNOS intracellular expression), both in lean and obese sedentary mice. Besides, only in obese sedentary animals, there were increases in anti-inflammatory cytokines (IL-10 and TGF-β) and decreases in pro-inflammatory cytokines (MCP-1, IL-8) expression after β2-adrenergic stimulation by terbutaline. This reflects a stronger, more dramatic switch to macrophage anti-inflammatory state in obese animals, involving not only phenotypic switch but also changes in the inflammatory activity profile. It is important to indicate that IL-8 and MCP-1 are two very potent chemokines, and particularly MCP-1 is the most important chemokine that regulates migration and infiltration of monocytes/macrophages [39], with a relevant pathophysiological role in the development of obesity and its complications [40]. According to our results, β2-adrenergic stimulation could contribute to reduce the local and systemic inflammatory state present in obesity. This is especially relevant considering that high levels of pro-inflammatory markers can also contribute to hypertension and insulin resistance in this condition [41,42].

Furthermore, it is also important to highlight that the results herein presented showed that the anti-inflammatory response of peritoneal macrophages to β2-adrenergic stimulation in obese animals is different from that occurring in healthy ones, being more intense in obesity. Therefore, these differential effects could contribute to improve the inflammatory state in obesity and its associated pathologies. Recent investigations from our group, carried out in monocytes from the same animal model of obesity, have also revealed that β2-adrenergic activation exerts differential effects in lean and obese sedentary mice, with anti-inflammatory effects taking place only in obese individuals, which presented a pro-inflammatory state at baseline [23]. Nevertheless, a potential risk of the anti-inflammatory effects (if not well regulated) is that they could induce immunosuppression of the innate function, further increasing susceptibility to pathogen challenge [26,27]. Previous results from our laboratory have shown that the phagocytic and microbicide activities of macrophages decrease in response to β2-adrenergic stimulation, both in lean and obese sedentary animals [28]. Therefore, the anti-inflammatory effects observed in the present work should be taken carefully and considering the potential innate response impairment that also takes place. Since pharmacological anti-inflammatory strategies such as β2-adrenergic agonists have been proposed for the management of this condition [18,19], it is critical to accurately determine the effects of β2-adrenergic stimulation in healthy and pathological conditions, especially when combined with non-pharmacological therapies such as physical exercise, when synergistic beneficial effects or undesired adverse effects can arise.

It has already been mentioned that physical activity is the most efficient non-pharmacological strategy in obesity and is used to control low-grade inflammation based on its potential anti-inflammatory effects [8,16,24,25]. However, intense physical activity can induce stress in obese individuals, causing inflammatory dysregulations that can exacerbate this pathology [9]. In peritoneal macrophages, it seems that the anti-inflammatory effect of terbutaline could be greater than that of exercise, since we have not found relevant changes with exercise alone in these cells. This is a different behavior than that occurring in circulating monocytes from the same animals, with anti-inflammatory effects both with exercise and with terbutaline in obesity [26]. Results in the present work indicate that β2-adrenergic stimulation in trained mice, after the 8-week regular exercise program (which leads to beneficial metabolic effects in lean and obese animals [26,28]), only causes a decrease in iNOS and IL-8 peritoneal macrophages expression both in lean and obese mice, thus reducing the percentage of macrophages with pro-inflammatory function in the peritoneal cavity. However, terbutaline-mediated increase in anti-inflammatory cytokines IL-10 and TGF-β, and ARG1+ macrophages that was observed in sedentary animals does not occur in trained ones. Consequently, exercise partially hinders the anti-inflammatory effect of β2-adrenergic stimulation, especially in obese animals. Previous studies carried out in our laboratory showed that this exercise protocol in the same mice strain decreased the expression of β2-adrenergic receptors in the peritoneal macrophages from lean and obese animals [28]. We could speculate that regular exercise-mediated lower expression of this receptor in peritoneal macrophages could be, at least partially, impeding a greater β2-receptor activation, so β2-adrenergic stimulation during this regular exercise program would not have a direct and dramatic phenotypic and activity profile anti-inflammatory effect on peritoneal macrophages (as in sedentary animals), but only reduce M1 macrophages. This can be, at least in part, an explanation for the lower anti-inflammatory effects of terbutaline in exercised animals. A recent study carried out by Lou and colleagues (2020) has shown that eccentric exercise accompanied by a low-fat diet rescued obesity-induced insulin resistance and improved exercise capacity, which were associated with the inhibition of M1 macrophage polarization and the activation of M2 macrophages, pointing out that macrophage polarization provides a potential target of intervention for inflammation and insulin resistance in obesity [37].

Then, it is plausible to think that while the effect of β2-adrenergic stimulation modifies the inflammatory profile of peritoneal macrophages in obesity (potentially contributing to improve related complications), when this strategy is combined with exercise, the effect is not amplified: on the contrary, it is weaker. Thus, before implementing regular exercise programs as an anti-inflammatory strategy for the treatment of obesity or other low-grade inflammatory pathologies, it is crucial to determine potential pharmacological or non-pharmacological interactions that could reduce the potential beneficial impact in the individual; according with the optimal intensity and duration of exercise programs in order to avoid potential undesired effects. Interestingly, previous results from our group showed that regular exercise can revert the inhibitory effect of terbutaline on the phagocytic activity of macrophages (although obesity seems to hinder this immunophysiological adaptation) [28]. Therefore, despite the anti-inflammatory effect of β2-adrenergic stimulation in trained mice is not as strong as in sedentary animals, it seems to be better regulated and does not immunocompromise the innate response.

Surprisingly, results showed that β2-adrenergic stimulation by terbutaline increased TNF-α expression in peritoneal macrophages in both obese and lean sedentary mice. Recent investigations from our laboratory in the same animal model showed that terbutaline-mediated β2-adrenergic stimulation in monocytes also tends to increase intracellular expression of TNF-α [23]. In accordance with this, it has been suggested that the response of inflammatory cells to catecholamines could stimulate the production of inflammatory mediators (including TNF-α) in inflammatory pathologies [35] such as obesity and obesity-associated diabetes [16]. Therefore, the global anti-inflammatory effect observed in sedentary lean, and most importantly obese mice, could be partially counterbalanced by this paradoxical increase in TNF-α. However, after the exercise protocol, the response of TNF-α in β2-adrenergic stimulated-macrophages is different in obese (expression decrease) and lean (expression increase) mice. Decrease in macrophages expressing TNF-α in obese mice is especially relevant since TNF-α is a very important cytokine in the pathophysiology of obesity and insulin resistance. In this line, a recent study carried out by Bu and colleagues (2020) indicated that the inhibition of TNF-α release from activated macrophages relieved insulin resistance in skeletal muscle, concluding that TNF-α might become a therapeutic target to attenuate and control insulin resistance in obesity and type 2 diabetes [43]. Nevertheless, these paradoxical TNF-α results should be taken carefully, since they do not follow the global behavior, which could also be a partial limitation to our interpretation of the global inflammatory effects. Even assuming the potential inaccuracies underlying any generalization, our conclusions are based on the global inflammatory behavior, which, in our opinion, is very important from an immunophysiological point of view.

## 5. Conclusions

β2-adrenergic stimulation causes a global phenotypic anti-inflammatory effect in lean and obese sedentary mice, which were more drastic (also including anti-inflammatory effects on the cytokine profile) in obese animals. In trained lean and obese animals, this anti-inflammatory effect is weaker and only evident by decreased iNOS and IL-8 expression. Thus, β2-adrenergic activation leads to anti-inflammatory effects, but these effects are, at least in part, modulated by obesity in sedentary conditions, as well as by regular exercise; but not by obesity in trained condition. It is also important to note that, due to the low number of animals used in the present study, as strongly recommended by the Bioethics Committee, an improved statistical power reinforcing the conclusions could be obtained with a greater number of animals in each experimental and control groups.

## Figures and Tables

**Table 1:** Body weight, dietary and energy intake and metabolic profile in obese and lean mice (sedentary and trained).

	Lean		Obese	
	Sedentary	Trained	Sedentary	Trained
Body Weight (g)	29.3 ± 1.2	25.6 ± 1 *	42.3 ± 1.15 •	36.0 ± 3.0 *
Dietary Intake (g/day)	4.0 ± 0.1	4.1 ± 0.1	2.7 ± 0.1 •	2.5 ± 0.03 *
Energy Intake (Kj/day)	55.3 ± 3.1	56.6 ± 0.9	62.01 ± 2.7 •	57.6 ± 0.7 *
Glucose (mg/dL)	218.9 ± 13.2	196.4 ± 25	311 ± 31 •	282.5 ± 28
Cholesterol (mg/dL)				
- Total Cholesterol	103.7 ± 2.2	106.7 ± 3	172.7 ± 19 •	178.1 ± 25
- HDL-C	42.1 ± 2.9	51.7 ± 4 *	59.7 ± 5.7 •	75.3 ± 4 *
- cLDL-C	50.7 ± 3.5	39.4 ± 2 *	88.8 ± 16 •	38.5 ± 1 *
Triglycerides (mg/dL)	86.8 ± 1.9	76.6 ± 1 *	91.5 ± 2 •	80 ± 1 *

Each value represents the mean ± SEM of the determinations (one per independent animal) in duplicate. * *p* < 0.05 with respect to the corresponding sedentary group value; • *p* < 0.05 with respect to the lean sedentary group values. HDL-C—high-density lipoprotein cholesterol; cLDL-C—calculated low-density lipoprotein cholesterol [28].
